# ‘Helper’ or ‘punisher’? A qualitative study exploring staff experiences of treating severe and complex eating disorder presentations in inpatient settings

**DOI:** 10.1186/s40337-023-00938-1

**Published:** 2023-12-07

**Authors:** Sienna Bommen, Helen Nicholls, Jo Billings

**Affiliations:** Division of Psychiatry, University College London, 6th Floor, Maple House, 149 Tottenham Court Road, London, W1T 7NF UK

**Keywords:** Eating disorders, Mental health, Staff wellbeing, Qualitative, Interview, Reflexive thematic analysis, Inpatient, Trauma

## Abstract

**Background:**

Eating disorders have been described as challenging to treat, with the most severe cases requiring inpatient admission. Previous studies have explored staff’s perspectives on eating disorders and service provision. However, little is currently known about how staff experience working with severe and complex eating disorder presentations in inpatient settings and how they may be impacted by their work.

**Aims:**

This study aimed to explore the experiences of staff who contribute towards the treatment of severe and complex eating disorder presentations in inpatient settings.

**Methods:**

Participants were recruited purposively via clinical contacts and a private hospital group in the UK. Semi-structured interviews were conducted, recorded, transcribed verbatim, and analysed guided by the principles of reflexive thematic analysis.

**Results:**

Interviews were completed with twelve staff members, including frontline nursing staff and multidisciplinary team (MDT) staff, from both private and public specialist settings. Participants expressed uncertainty about the treatment approach for service-users with severe and complex presentations. As service-users often resisted treatment, participants felt positioned as a ‘punisher’ rather than a ‘helper’ in initial treatment stages. Many had experienced physically and emotionally impactful events at work, including violence and aggression, as well as serious incidents of self-harm and suicide attempts. Participants generally found support in their colleagues, however considered organisational support insufficient.

**Conclusions:**

This research highlights an urgent need to consider the wellbeing of staff who works in eating disorder inpatient settings, as well as their support needs. Retention and recruitment strategies should be reviewed to reduce staff turnover and improve patient care. Further research should investigate whether specialist inpatient staff are impacted by symptoms of vicarious or direct trauma, moral injury and compassion fatigue.

**Supplementary Information:**

The online version contains supplementary material available at 10.1186/s40337-023-00938-1.

## Abstract (plain language summary)

This study explored how staff who treat severe and complex eating disorders experience working in inpatient settings. The researchers interviewed twelve staff members from inpatient eating disorder services based throughout the UK. The themes identified from the interview data described challenges related to treating people under the Mental Health Act (without their consent) and the dynamics that arise in this context – staff often found it hard to be seen as ‘the punisher’ when delivering treatment, as it felt like a contradiction to their career in helping others. Staff described physically and emotionally taxing work and their reliance on co-workers in coping with this, as they found organisational support insufficient. Several staff described experiencing directly and indirectly (vicariously) traumatising events at work. This highlights an urgent need to investigate trauma responses in the context of inpatient work with severe and complex eating disorders, as this will enable organisations to implement appropriate staff support. This is likely to in turn improve patient care.

**Supplementary Information:**

The online version contains supplementary material available at 10.1186/s40337-023-00938-1.

## Background

Eating disorders are serious psychiatric disorders, that require extensive treatment, including inpatient admissions for severe cases. Among the inpatient population, Anorexia Nervosa (AN) is most commonly observed [11]. The lifetime prevalence rates of AN are estimated to be up to 4% among females and 0.3% among males [33]. The disorder is characterised by a fear of weight gain and a drive for thinness, which is achieved through restrictive eating and compensatory behaviours like exercise, self-induced vomiting, or laxative abuse [35]. Serious medical complications are common due to severe weight loss and excessive engagement in the behaviours associated with the disorder [26]. Mortality rates are high, especially in the inpatient population [2], with deaths usually resulting from medical complications or suicide [16].

Treating AN or similar eating disorders is multifaceted and a multidisciplinary approach is therefore recommended [17]. Evidence-based treatment of AN usually involves medical stabilisation, pharmacological treatment, nutritional treatments, family interventions for young people and tailored eating disorder psychotherapies for adults [35]. Nasogastric feeding (NG-feeding) is sometimes required due to food and/or fluid refusal, and at times NG-feeds are carried out with physical restraints against the will of the service-user under the Mental Health Act [14]. Ambivalence about pursuing recovery and treatment non-compliance are common factors of AN treatment [1], which has sparked ethical debates about consent and mental capacity [13].

There is a lack of an empirically derived definition of the more severe, complex and/or enduring eating disorder presentations, which means we know less about effective treatment and management of these cases [21]. This is despite psychiatric comorbidities having been found to be very common in sufferers of AN and Bulimia Nervosa [25, 29]. According to Woodside and Staab [34], the lifetime prevalence of comorbid obsessive–compulsive disorder (OCD) is estimated to be around 40%, social phobia 20%, substance abuse 17%, specific phobias 15%, post-traumatic stress disorder (PTSD) 13%, panic disorder 11%, and generalised anxiety disorder 10%. A large proportion of people with AN also meet diagnostic criteria for “personality disorders” and have complex emotional needs [24].

Traumatic backgrounds are commonly reported by people with AN (Mitchell et al., 2012) and may contribute to a particularly complex eating disorder presentation [6]. A study by Longo et al. [22] found that relational traumas were most common in people with AN, and that the eating disorder symptoms of those with comorbid PTSD symptoms differed from those without PTSD symptoms. A quantitative study found that early trauma compromises treatment outcomes of AN due to the role of emotion dysregulation in this group [8]. It is unknown whether rates of diagnosable trauma-disorders in people with eating disorders may increase with the introduction of the diagnosis of Complex-PTSD in the ICD-11 [23].

Due to the complexities and risks associated with eating disorders, contributing to treatment is likely to engender unique experiences for specialist eating disorder clinicians. This may be particularly pertinent in inpatient settings, where staff are working with the most severe and complex presentations. To date, very little research has focused on the specialised area of experiences of staff who treat severe and complex eating disorder presentations in inpatient settings. A qualitative study by Davey et al. [12] explored work demands and social support for nursing staff and occupational therapists in eating disorder inpatient settings. This study reported good patient-related job satisfaction, while organisational factors like admin work, staffing resources and communication difficulties were felt to be the primary sources of dissatisfaction. A qualitative study by Reid et al. [30], explored staff views of eating disorder treatment in the UK and highlighted further concerns about practical and organisational issues. Participants considered the patient group diverse and felt the patient needs were not adequately met. Lack of training for staff and a consequent feeling of being unable to help the patient group caused frustration, impeded therapeutic relationships, and thereby reduced perceived efficacy of treatment. Participants voiced a need for specialist services to respond to patient needs.

Specialist inpatient units require high-risk tasks of professionals. Kodua et al. [20] conducted a qualitative study exploring nursing-assistants experience of completing compulsory NG-feeds with manual restraints (physical holds without equipment such as belts or cuffs). Participants described the experience as distressing, some used the word ‘traumatising’, and reported frequent physical exhaustion, physical injury and physical aggression from service-users.

To date, little is known about how staff in specialist services may be impacted by working with some of the most complex and high-risk patient groups in psychiatric care. This research aimed to address this gap. Learning more about the impact this work can have on staff may contribute towards informing organisational support and training, as well as recruitment and retentions strategies. This may in turn improve the quality of patient care. Therefore, this study specifically aimed to explore how staff experience contributing towards treatment of severe and complex eating disorder presentations in specialist inpatient settings, with an emphasis on the impact of the work on the staff and how they cope.

## Methods

### Ethics

Ethical approval was awarded by University College London Research Ethics Committee (Project ID: 23073.001) (Additional file 1: Supplementary material 1).

### Participants and procedures

Participants were recruited purposively in two ways: through clinical contacts of the research team and through a private hospital group that specialises in eating disorder care. Further participants were recruited by inviting existing participants to share the study with potentially suitable colleagues. Email and social media were used to inform clinical contacts about the study. The research team aimed to recruit a range of professionals, with a range of length of practice, to increase the diversity of the results. All participants had to have experience of working with severe and complex eating disorder presentations in inpatient settings.

Interested volunteers were invited to email the research team and were then sent the participant information sheet and consent form via email. No participants dropped out, and all gave fully informed written consent. Participants were given the option of face-to-face interview or remote interview.

The interviews followed a semi-structured format. The interview topic guide (see Additional file 1: supplementary material) was collaboratively developed in advance by the research team and a small number of multidisciplinary staff from the private hospital group, who have lived experience of providing specialist inpatient eating disorder care. Interviews were recorded and transcribed verbatim by the interviewer, using NVivo followed by manual correction. Any potentially identifying information about the participant, their place of work, or service-users was removed from interview transcripts. Pseudonyms have been applied to the participants.

### Analysis

Transcripts were analysed using reflexive thematic analysis (TA), guided by the six principles of TA developed by Braun & Clark, (2019). Familiarization with and immersion in the data included reading and re-reading transcripts. Inductive organisation of participant quotes and semantic codes was completed to gain an overview of the prominent content across transcripts. Latent codes were then searched for and organised. Connections between codes were searched for, which enabled higher order inductive themes to be generated. Subthemes were explored and generated, before themes and subthemes were reviewed, re-organised, defined and named with the specific research question in mind. Reflexive notes were taken throughout this process, with the position and experience of the interviewer and research team in mind. We aimed to increase credibility of our study by presenting some of the participants with our findings and reflecting on whether it felt as though we encompassed their experiences. We aimed to increase validity by presenting a draft of our research paper to a small focus group from the private hospital group we recruited from. The focus group did not partake in interviews and as such their resonation with the findings demonstrated further face validity of the findings.

### Ethical issues

The research team was mindful about the sensitive nature of some of the experiences that clinicians may choose to talk about. Signposting to support organisations was included in the participant information sheet and a clear safeguarding protocol was developed before interviews commenced. We offered flexible interview times to accommodate shift patterns and work demands. We also aimed to recruit widely across services and professional roles, with the intention of not placing pressure on specific teams or services. Participants were provided with a £20 e-voucher to thank them for participating and as compensation for their time.

The research team themselves were supported with training and supervision in response to the potential impact of hearing about mental health professionals’ distress, especially as some of the experiences raised by clinicians were similar to those of the research team.

### Quality

Concepts that demonstrate rigour and quality in quantitative research, such as reliability and generalisability, have been widely critiqued as inappropriate for qualitative research [31]. Therefore, the research team aspired to fulfil quality criteria relevant to qualitative research, such as trustworthiness [28]. To do this, we used existing frameworks to guide us at each phase of our research process and analysis, such as Nowell et al. [28] and Braun & Clarke [4].

In addition to credibility and validity checks, we have made efforts to be open and specific about our decision making, the steps we have taken to generate our themes, and the potential impact of the background of the researchers. This contributes towards increasing transferability, should other researchers wish to replicate the study. We have established dependability as a traceable process is documented. Confirmability was sought by demonstrating that data is clearly derived from the data through our inductive codes, as well as use of verbatim quotes from the transcripts that demonstrate our arguments.

### Reflexivity

The research team consisted of three white women from working- and middle-class backgrounds.

S.B is a MSc student on a masters level clinical mental health sciences course at the time of the study. She has worked in a private specialist eating disorder inpatient service for over four years, in three different roles, including Healthcare Assistant and Assistant Psychologist. The latter was her employment role at the time of the research.

J.B is a consultant clinical psychologist and professor with over 23 years of experience of working in the NHS with specialist expertise in trauma, mental health and well-being in high-risk occupational groups.

H.N is a doctoral student with experience in qualitative research in healthcare settings.

The research team’s experience of specialist clinical work and related settings was advantageous, particularly for S.B who conducted the interviews, as this experience contributed towards a shared understanding between interviewer and interviewee. S.B.’s work experience positions her as an ‘insider’ of specialist inpatient care, as a previous frontline worker and current multidisciplinary team member. She can also be viewed as an ‘outsider’ by frontline staff as a current multidiciplinary team member or by public sector staff as privately employed. This may have influenced, positively or negatively, the participants’ level of comfort or openness in sharing stories about their work.

There are potential disadvantages in our closeness to the research area. We were mindful of a ‘pull’ towards statements we personally resonated with. This was addressed throughout all stages of data collection and analysis by taking a curious approach to our data and using the whole research team to search for potentially overlooked themes or perspectives. We also conducted credibility and validity checks with participants and other professionals with lived experience of working in similar settings.

## Results

Twelve mental health professionals participated in the study. The gender, ethnicity, professional role, work setting, length of work in specialist eating disorder care and geographical locations of employment are shown in Table 1.

**Table 1:** Demographic information of participants in the present study

Characteristic	*N (Total* = *12)*
*Gender*	
Female	9
Male	2
Other	–
*Age*	
18–24	–
25–34	4
35–44	4
45–54	–
55–64	–
65–74	–
74 +	–
*Ethnic group*	
White British	4
Any other white background	3
Caribbean	1
Prefer not to say/not reported	1
Length of time in mental health care	
Less than 1 year	–
1–2 years	2
3–5 years	3
6–10 years	3
11–20 years	–
21–30 years	–
30 + years	–
*Length of time working with severe and complex eating disorder presentation*	
Less than 1 year	2
1–2 years	2
3–5 years	5
6–10 years	–
11–20 years	–
21–30 years	–
30 + years	–
*Geographical area*	
London	5
Midlands	1
North East	1
North West	–
South West	–
Scotland	–
Wales	–
Northern Ireland	–
*Currently working in inpatient ED setting*	
Yes	7
No	2
*Core profession/role*	
Family Therapist	1
Nurse (and senior nursing roles)	2
Healthcare Assistant/Support Worker	3
Occupational Therapist	1
Dietician	1
Consultant Psychiatrist	1
Psychologist	1
Assistant Psychologist	2

Most participants were working with service users with severe and complex eating disorder presentations in an inpatient setting at the time of the interview. Some had left inpatient care to work in other healthcare settings.

Interviews were completed between the 2nd and 25th of August 2022. Interviews lasted between 17 and 70 min, with most interviews lasting between 30 and 45 min.

## Overview of themes and subthemes

Three themes were derived from the data, each with three subthemes. These describe staff’s experiences of treating severe and complex eating disorder presentations in inpatient settings in the UK, as well as the impact the work has on staff and how they cope (see Table 2).

**Table 2:** Themes and subthemes identified in interviews with staff from specialist eating disorder inpatient settings:

Theme 1: *‘The Punisher’* – Delivering treatment against consent
a. From ‘h*elper* ‘to *‘punisher’*
b. Helping or hurting? Uncertainty about treatment approaches for complex presentations
c. *‘It’s bloody traumatising’ *– The impact of contributing towards treatment on staff
Theme 2: *‘The Abused’* – Casualties of the system
a. Violence and aggression when the eating disorder fights back
b. *‘Bottom of the ranks’* – *'no one cares about you'*
c. Insufficient support and training
Theme 3: Nurturing *‘a core of hopefulness’* – Coping with the work
a. Managing your mindset and ‘holding hope’
b. The *‘virtuous cycle’* of relational work
c. *'Listen and take action' *– what clinicians need to maintain wellbeing at work

## Theme 1: *‘the punisher’* – delivering treatment against consent

### Subtheme 1.1 from ‘helper’ to 'punisher’

Participants described high levels of distress as a result of delivering compulsory treatment, for both service-users and staff. The sense of causing distress in the service-users, even with the knowledge that the treatment is often lifesaving or necessary, was experienced as emotionally challenging.

“Having these, these kids sit there and scream and beg and cry for you to, you know, to not make them eat their sandwich, to not put that tube down them and still having to do it is… It's hard. It's really, really, really hard.” – Amber, Nurse.

Participants described eating disorders as having a function for the service-users, and as such, the service-users may desperately want to hold onto their behaviours. Several participants reflected on how it can feel like they are being positioned as a “punisher” when needing to reinforce rules or treatment procedures without their consent. This is demonstrated by the following quote from a healthcare assistant:

“(…) they are incredibly defensive of their illness whilst at the same time slightly hating that illness, so it means that they will go to the ends of the earth to defend their illness. And that means that quite often you become the oppressor almost—if you're trying to get them to fight against the illness, you then become the enemy. You become the… the abuser or the oppressor. (…) we all want to feel appreciated for work that we put in so that it can feel really frustrating when you're trying literally everything within you to help someone. And it feels like you're just getting resistance, defensiveness, aggression, abuse, it's like, it's very troubling.” – Liv, Support Worker.

## Subtheme 1.2 Helping or hurting? Uncertainty about treatment approaches for complex presentations

Participants understood severe and complex eating disorder presentations as very diverse conditions. Participants mentioned several comorbidities, most commonly trauma-related disorders or complex emotional needs, as well as frequent co-occurrence of autism. Several participants spoke about not feeling skilled or trained enough to support service-users adequately, as illustrated by the below quote:

“We're seeing more and more trauma related kind of eating difficulties, which is very hard. We're not—we don't get a lot of training in trauma (…) I didn't feel like I ever had enough training for the—what we were being asked to do on the ward”. – Amber, Nurse.

The participants understood complexity as requiring a creative approach to treatment. Several participants described enjoying a creative approach and ‘*thinking outside the box*’. However, due to the high level of need and complexity, staff felt like they worked outside their expertise at times. This quote by a clinical psychologist illustrates this perception of working with complexity:

“Sometimes it's nice because you're learning new things. (…) You try different things to see if it works. But you can feel de-skilled because you don't know what's happening with this person. So, yeah, that can be really hard. And I think sometimes people thinking, as a psychologist, you're going to know how to work through all these things.” – Emma, Clinical Psychologist.

Participants explained that service-users may be prescribed less calories to encourage compliance with an oral diet as opposed to being NG-fed. Participants thought this may reinforce calories as something to be avoided. There were further concerns about whether the focus of weight restoration and achieving an oral diet minimises the importance of treating deeper issues or comorbidities. This quote from a dietician illustrates some concerns about this treatment approach:

“It just feels like a short-term fix to say, well,’ if you don't eat…’, you know, it's just a little bit threatening. Where you know, this young person, probably from a young age, has been treated in that sort of way and feel they have no control over their lives (…) I’ve not seen it really work.” – Vanessa, Dietician.

Other participants also worried about whether aspects of treatment may emotionally harm or traumatise service-users. This was especially true for nursing staff who carried out NG-feeds, as well as invasive interventions like intramuscular injections. This nurse spoke about realising that treatment may cause trauma:

“I think sometimes it hits me how much trauma I've caused to young people just by being in hospital.” – Amber, Nurse

Similarly, therapists struggled with some service-users who are ‘very silent’ or resistive to therapeutic interventions. In attempts to help and engage the service-users, MDT-staff described uncertainty about whether what they talk about makes sense to the service user or whether it may make things worse or ‘contributes to their trauma’.

*“I think one of the things that I find really challenging is when young people are very silent or very resistant to the idea of therapy because it leaves me with a real dilemma of not knowing how helpful it is for me to keep showing up, you know, whether I'm just sort of contributing to their trauma in effect or on the other hand, when they're very silent again, that's hard for me because I don't know whether I am saying things that make sense to them, whether I'm saying things that make things worse.”*

– Tatiana, Family Therapist

### Subtheme 1.3 ‘It’s bloody traumatising’ – The impact of contributing towards treatment on staff

Manifestations of distress, including self-harm and suicide attempts, were experienced as emotionally upsetting by staff. Due to the level of responsibility experienced within their roles, staff worried about making mistakes with potentially devastating consequences. Staff were aware of high mortality rates within their field, and as such, feared they would experience the death of a service-user. The below quote speaks to learning about the deaths of service-users:

“I haven't had anybody die that I've worked with directly, but there's people that, you know, they've been in your group, or they've been in services for a long time and then they die. And that's hard.” – Emma, clinical psychologist

Most participants spoke about the nature of the work impacting their wellbeing or mental health. One participant used anti-depressants and considered work a contributing factor. Others described crying at work and after work, feeling ‘completely exhausted’ and taking sick leave due to the impact of the work. It appeared junior staff were impacted more severely as described by this experienced clinician:

“But then I think you can see (…) new HCAs: they are petrified, they cry. They get tearful. They need even extra support because they've never seen that before.” – Natasha, Occupational Therapist.

Several participants with frontline experience highlighted the experience of prioritising the safety of service-users while being under attack from them. Some participants spoke of this in the context of trauma. This participant describes how staff ‘can’t escape’ when restraining aggressive service-users:

“You can't escape a hold, they can punch you, but you can't let go. (…). You can't escape. There were times  that you can't even use the toilet. You can't escape their verbal abuse in that moment. So for me, it's, it's, it's trauma.” – Marie, Assistant Psychologist.

## Theme 2: *‘the abused’* – casualties of the system

### Subtheme 2.1 Violence and aggression when the eating disorder fights back

Due to the service-users’ high levels of resistance to treatment, staff had experienced violence and aggression in the workplace. No participants described intent to harm from service-users. Rather, there was an understanding of violence and aggression as ‘push back’ from the eating disorder when it was challenged. Risk of injury had become routine for many frontline workers. This participant describes a particularly challenging incident, when a service-user physically fought the supportive holds that were implemented to avoid harm to the service-user:

“And she really, really dealt with me. She gave me all the kick, all the head, and everything just to stay away from her. Hit me and everything. (…) In the moment I couldn’t feel the pain, didn’t feel anything, it’s just me keeping her safe in that moment, but I stayed away for a week because I had bad injuries. And that’s how I ended up with this knee injury and some bruises on my legs and stuff like that, I was coming to this place with bandages all over my ribs that no one knew about it for a week.” – Colin, Support Worker.

As a result of violence and physical interventions at work, participants reported to have acquired short-term and long-term injuries. There were also reports of exacerbated pre-existing conditions, both physically and mentally. This quote illustrates how experiences at work can trigger pre-existing, personal experiences:

*“But it feels - it feels like you're being attacked, it feels personal, it feels so personal. And that for me personally is quite triggering just because of my, my own life, and my own personality is; if I feel attacked and I feel like someone's making me feel like I'm doing something wrong, then I automatically feel really upset and really... Despondent and want to give up.”*

- Liv, Support Worker

Supporting service-users with high levels of resistance to treatment impacted staff’s ability to maintain their compassion in times of stress or when feeling unsupported in their roles. Some worried about reductions in their empathy as they became accustomed to high levels of distress and resistance in the initial stages of treatment. The emotional impact of the work also transferred to private life. Participants felt exhausted and drained, with little energy to tend to private relationships, self-care and hobbies. This participant explains how the work impacted private relationships:

*“So that definitely has issues where I'd come home and I'd be in a mood. So you have a massive argument, like in terms of that, that would cause issues because, yeah, so you're constantly stressed and overworked”—Amber, Nurse.*

## *Subtheme 2.2 ‘Bottom of the ranks’* – *'no one cares about you'*

There was a consistent frustration among most frontline staff members about the lack of acknowledgement about the challenging nature of the work from management or senior staff. Some nursing staff expressed feeling taken advantage of, as they carry out mentally and physically risky work over 12-h shifts, with salaries that cannot compensate for their efforts or enable them to access the care they need due to the impact of the work.

*“It's like you are the least of priorities when you do the hardest job.”*

– Marie, Assistant Psychologist (about nursing roles)

Several participants, both frontline and MDT staff, described constant or fluctuating periods of time characterised by ‘blame culture’ and criticism. This impacted the team’s ability to work together.

*“I think when an organization has a culture of blame. That is one of the most toxic things that we can be doing to each other because it creates so many things along with it suspicion, mistrust and pressure and that really, really shuts down people's ability to think and do their jobs. So one of the things that, for example, is really helpful for me is that I feel safe. I feel surrounded by people who respect me enough to either help me or at the very least, stay out of my way and let me do my job, so that's a really nice feeling because it allows that sense of agency and freedom. So when you experience the opposite; of people kind of really breathing down your shoulder or questioning everything that you're doing or telling you that you're doing things wrong, that's something that's kind of difficult to withstand.”*

–Tatiana, Family Therapist

Participants interpreted the lack of support and insufficient salaries as a sign of disrespect. This felt detrimental to work satisfaction, as highlighted by this quote:

*“I mean, I like the patients here. But it’s the support we don’t have. That’s what I mean. The support, the lack of money and the lack of respect here. They don't respect us.”*

– Alicia, Support Worker

### Subtheme 2.3 Insufficient staffing levels, support and training

The stress and high vigilance required at work was further complicated by the consensus that staff turnover and insufficient staffing numbers increased risks. This placed even further responsibility on the staff who were familiar with the service-users. This nurse spoke about two difficult incidents preceding her change in employment, that highlights her role in decision-making about service-user safety and how reading the cues of one service-user potentially saved their life:

“(…) Then I had two serious incidents in the week of self-harming. I would turn to somebody who needed, I don't know, God knows how many stitches, and I've dealt with self-harm incidents, but there was so much blood and profusely and heavily. And I, had I not been quite so busy that morning, I think I should have taken different action, I should have maybe increased her level of obs, but we were trying to avoid doing that. Oh, I don't know. (…) After that, I was a bit twitchy because of what happened the previous day and I just had a bad feeling about the patient, and I went in, and there was no response, I found her purple having tied a ligature. (…) If I hadn't have had the incident on the previous shift—if I hadn't been a regular staff member on my shift…”—Cornelia, Nurse.

Staff pointed out that ‘one patient may be like treating two or three patients’ due to the high needs. Participants felt there was a correlation between acuity and sickness, as staff struggled to cope when the ward had disproportionate numbers of service-users who require very challenging treatment. Participants felt that complex and severe eating disorder presentations require higher staffing levels due to the intensity of the work. Importantly, participants noted that the skills and experience of the staff also mattered to distribute the work evenly across the team, as described by this healthcare assistant:

“A lot of folks go through injuries, stress and all this. At the end of the day, it’s a 12-h shift. A 12-h shift. Each hour is a very, very intense stress. (…) Imagine you’ve been in restraint for two or three hours. Coming from one restraint, going to the next restraint. Just like that. Just like, because of, because maybe you have 20 staff on, but it's only X staff that is capable of doing everything because the other ones can't physically handle what you see.“

– Colin, Support Worker

Resolving staffing difficulties minimised the time available for focusing on service-user needs and safety, as described by this nurse:

*“Just looking on the rota and thinking: ‘right - there is me and maybe one other person that I know is regular, and then all the rest is completely brand new’ - and that's going to take up a lot of time at the beginning of the shift, just when I need to be thinking about what the patient's needs are and thinking about staff to make sure that we can have a safe shift and then just worrying that something will slip through the cracks”*

- Cornelia, Nurse

### Theme 3: nurturing *‘a core of hopefulness’* – coping with the work

#### Subtheme 3.1 Managing your mindset and ‘holding hope’

Staff reported to cope with the difficulties of their work by adjusting their mindset and expectations. During the difficult initial stages of treatment, participants could endure the challenges as they knew it was temporary and necessary.

*“I need to prepare my mind, like; this is not always going to be like this. (…) I get that at the moment that person is really unwell, so I need to, in a way, train myself or use positive affirmations with myself in order to keep me going.”– Natasha, Occupational Therapist*

Participants spoke about coping with the initial stages of treatment, when treatment appears most upsetting to the service-users, by holding hope for the future. Staff with more experience were able to remind themselves of previous positive outcomes. Junior staff described moments of doubt and dwindling hope, due to the few and far between signs of progression. Senior staff were named as supportive and helpful in reigniting hope, as described by this healthcare assistant:

“Once I, like, burst into tears to my boss and was like, ‘nothing is changing. It's just the same. No one's going to get better. It feels like they're all going to be like this forever.’ And she gave me so many examples of patients who are in the same situation, if not worse, who had managed to recover. And so that was helpful to know that it's happened in the past. It can happen again.” – Liv, Support Worker.

There was a sense of slow progression among the participants, which at times could feel difficult. Staff coped with this challenge by adjusting their expectations for recovery. As rewarding as the few instances of observed full recovery could be, the norm was that full recovery may not be seen while the service-users are in specialist inpatient settings. Participants coped with this by reminding themselves about the importance and value of their work. Some participants reported to feel able to celebrate each sign of positive change, whether small or big.

## Subtheme 3.2 The ‘virtuous cycle’ of relational work

Complex and severely unwell services users were experienced as challenging to communicate with at times. Staff reported to use significant personal resource and energy to ‘reach’, or connect with, the service-users. Deep reflections were required to consider how to be a resource to the service-users. When successfully overcoming communication challenges, by establishing a meaningful connection, staff found it incredibly rewarding. This participant explains how overcoming relational challenges creates a virtuous cycle of reward that acts as a protective factor in the face of adversities:

“So as challenging as I find reaching, trying to reach somebody who doesn't want to be reached or seems very closed—to be able to find a way through and figure it out is a fantastic feeling. (…) When I feel (…) like I've made good relationships with families or feeling that I've had a positive impact, whether it's on a family or a young person or a team; again, that becomes a virtuous cycle for me because the, that sense of reward helps me to keep going through the tough things” – Tatiana, Family Therapist.

Staff enjoyed the relational aspects of their work, which was possible due to the ‘longer than usual’ inpatient stays, as staff were able to get to know the service-users very well. Participants from both sectors enjoyed getting to know the service-users over time and appreciated the uniqueness of the long-term therapeutic relationships. This healthcare assistant spoke about their appreciation of the setting and the service-users as multifaceted and unique people:

*“I don't think that's a very common job where people; where your job; is making like very personal connections. It's incredible because it just gives you such an appreciation for, like, every individual person and how different and also similar people are in their talents, their sense of humour, their brilliance, their ambitions, their goals, the things they care about. So it's just so nice that I got to like, see that every day.”*

- Liv, Support Worker

### Subtheme 3.3 'Listen and take action' – what clinicians need to maintain wellbeing at work

A need to be heard and listened to, with appropriate action as a response, was important for participants across all roles. There was a felt frustration about ‘*the managers of our managers’,* the people who control or influence admissions, regarding the extremely high levels of needs on the wards. This participant described how they wish leaders would have more insight into this:

*“So something that it would help is for the managers of my managers, is to be able to have a better insight that one bed is not equal to the intensity that we are going through. Sometimes one patient that occupies one bed is equal three beds, because you have to spend a lot of time. (…) What is not working is the fact that no matter if we are to say, ‘this is very acute at the moment, we are not coping’, they are still adding people at this level of acuity.”*

– Natasha, Occupational Therapist

Participants across professional roles valued a united team, in which all feel able to express their views and influence decision-making. One participant spoke of this in the context of how the eating disorder psychopathology can be replicated in the ward environment:

“The eating disorder psychopathology is one of sort of quite extreme control and restriction. And then that can be echoed in the ward environment as in everything can feel quite controlled and frightening and quite hard to change plans and things. I say anything which helps to de-escalate that a little bit is useful. So for example, trying to make sure that all team members have the opportunity to express their views and to have that reflected in management plans so that colleagues don't feel like they are being imposed upon without autonomy or be forced into this like ‘punisher’ role without, you know, without having any control over that.” – Sam, Consultant Psychiatrist

Participants wanted more ‘thinking space’ to reflect and process their experiences. Junior staff appreciated contact with senior clinical staff for learning and reassurance. It was important for staff to understand the rationale behind treatment as well as being able to have their voices heard in the creation of treatment plans. Participants highlighted reflective supervision, reflective practice and training as useful to inform their work, contextualise their experiences and grow as professionals. Some participants had very rare supervision, which leaned more towards managerial supervision rather than reflective and clinical supervision. This was experienced as tokenistic and unhelpful.

“I think we need to have thinking spaces but also thinking spaces that feel accessible and that work—if we have, you know, a tokenistic supervision and actually the people who really wants to be there can't be there, that's a wasted space.” – Tatiana, Family Therapist

## Discussion

The present study aligns with previous findings regarding eating disorder inpatient staff’s patient-related job satisfaction and organisational dissatisfaction [12]. The participants in the present study derived job satisfaction from colleagues, collaboration across professional disciplines and the personal and professional growth they experienced in their roles. In terms of working with severe and complex presentations, we found that participants enjoyed that the long-term hospital stays enabled them to build strong therapeutic relationships with service-users. Supporting service-users and seeing progress was experienced as very rewarding as it required hard work. Participants also enjoyed the diversity of treating ‘body and mind’ and the creativity involved in treating complexity. Sources of dissatisfaction were largely in relation to lack of support, managerial attitudes, a culture of blame and low salaries/benefits.

In accordance with findings from Kodua et al. [20], participants highlighted NG-feeds as very distressing and potentially traumatising. The findings of this study elaborated on staff’s experience of their role in treating individuals who are highly resistive. Participants in this study acknowledged the distress of the service-users during this procedure, and having pursued helping-professions, they struggled with feeling positioned as a ‘punisher’ when partaking in distress-inducing treatment procedures without consent, including NG-feeds.

Participants reported they frequently witnessed high levels of distress, self-harm and suicide attempts. Several staff expressed a fear of making mistakes, as they were aware of the potentially deadly consequences. Participants also experienced violence and aggression at work in the context of service-users resisting treatment. Physical violence resulted in injuries for several participants and verbal aggression had an emotional impact. Many participants spoke about potential workplace trauma and described several concepts resonant of those that appear in recent occupational and vicarious trauma literature, such as moral injury, betrayal trauma, vicarious trauma, and compassion fatigue [3, 10].

Some staff, particularly frontline staff, felt disrespected, unappreciated and under-valued due to low salaries and managerial attitudes. There were also reports of ‘blame culture’. Participants from both private and public settings, particularly frontline staff, felt taken advantage of. They explained that due to high staff turnover and frequent use of new agency staff, permanent staff were given extra work demands, which resulted in further exhaustion. Additionally, participants connected scarcity of skilled staff familiar with the service and higher acuity on the ward to higher levels of sick leave and greater risks to service-users. These findings aligned with the organisational dissatisfaction reported in Davey et al. [12].

Participants did not feel adequately supported with the physical and emotional impact of their work. A shared understanding with the team appeared to be the primary source of support at work. Participants considered ‘thinking space’ such as supervision and reflective practice important, but the offer of this was scarce and often perceived as tokenistic or not focused on wellbeing. Several participants did not feel heard about their struggle with high acuity on the ward and expressed a wish for a greater appreciation about the intensity of treating severe and complex presentations across levels—from service managers to the ‘managers of the managers’.

## Strengths and limitations

To our knowledge, this is the first study to explore staff experiences of partaking in the treatment of severe and complex eating disorder presentations in specialist inpatient settings, across the public and private sector. This study included staff from one private hospital group and several public healthcare settings in the UK, as well as both frontline and MDT staff, which increases the diversity of the experiences reported. It also provided insight into the similarities of experiences across the private and public sector. The professional background of the research team provided a further strength. The research team had experience working in inpatient care and researching occupational wellbeing. S.B. had over four years of experience working in a specialist eating disorder inpatient hospital, employed both in nursing and multidiciplinary roles. When conducting interviews, this enabled a shared understanding to be established with participants, while remaining curious about details of the experience of staff in different roles and services. Rigorous methods of qualitative analysis, such as validity and credibility checks, add to the strengths of this study.

The findings of this study should also be considered in the context of its limitations. In terms of our sampling method, there is a possibility that disseminating details of the study via the research team’s professional social media accounts and inviting participants to pass on details of the research to colleagues (as well as through posters in the private hospital group) might have led to participation from clinicians who shared certain characteristics, which may mean their experiences are more similar than within samples collected by different methods. Participants who completed the demographic form were relatively early on in their careers and primarily from London or the south of England. Staff from other geographical locations or with additional years of practice may have different experiences to what is described in this study. There was also little ethnic diversity within our sample, partly due to incomplete demographic information sheets being filled out by participants. Minority groups may have unique perspectives that are not accounted for in this study.

## Conclusions

This study highlights a need to monitor the physical and emotional wellbeing of inpatient staff who contribute towards the treatment of severe and complex eating disorder presentations, due to the high level of manifestations of emotional distress staff are exposed to, the nature of the clinical treatment and perceived lack of support. Staffing level policies should be reviewed in specialist units in which staff experience high physical and emotional demands, to spread demands more evenly and mitigate risks of burnout. Findings from the present study can be utilised to inform retention and recruitment strategies, staff wellbeing initiatives and risk management, which may in turn improve patient care. Retention of staff appears to be particularly important, as experienced staff describe a higher level of resilience, confidence in safely managing complex presentations and consistency of care. Improved quality of care through staff wellbeing may improve treatment outcomes and lengths of stay.

Future studies should urgently examine the presence of compassion fatigue, moral injury, vicarious and direct trauma in specialist eating disorder inpatient settings. This can help assess risk accurately and implement preventative measures as well as appropriate occupational support. Future studies could also explore the unique experience of different professional disciplines, such as healthcare assistants, dieticians, nurses, psychologists or psychiatrists to explore more tailored support needs.

### Supplementary Information

**Additional file 1.** Ethics and Interview topic guide.

## Data Availability

Data has not been made publicly available due to the personal and sensitive nature of participant interviews and in line with the ethical approval for this study.

## Abbreviations

AN: Anorexia Nervosa
MDT: Multidisciplinary team
N: Number
NG: Nasogastric
OCD: Obsessive compulsive disorder
PTSD: Post traumatic stress disorder
