# Supplementary material for: ‘Helper’ or ‘punisher’? A qualitative study exploring staff experiences of treating severe and complex eating disorder presentations in inpatient settings
Source: J Eat Disord. 2023 Dec 7;11:216. doi: 10.1186/s40337-023-00938-1 (PMC10704651; doi:10.1186/s40337-023-00938-1)
Supplement: Supplementary file 1 — Additional file 1. Ethics and Interview topic guide. [file 40337_2023_938_MOESM1_ESM.docx]

Supplementary material 1


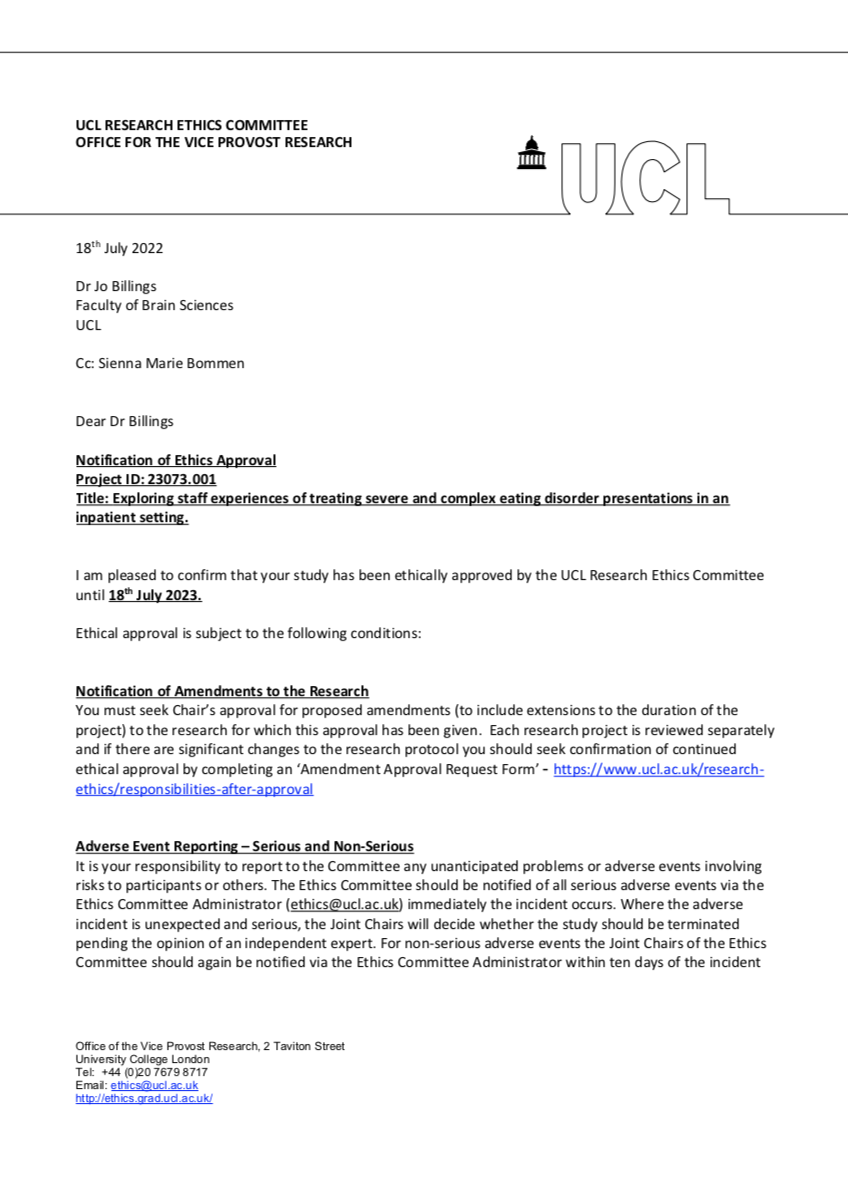


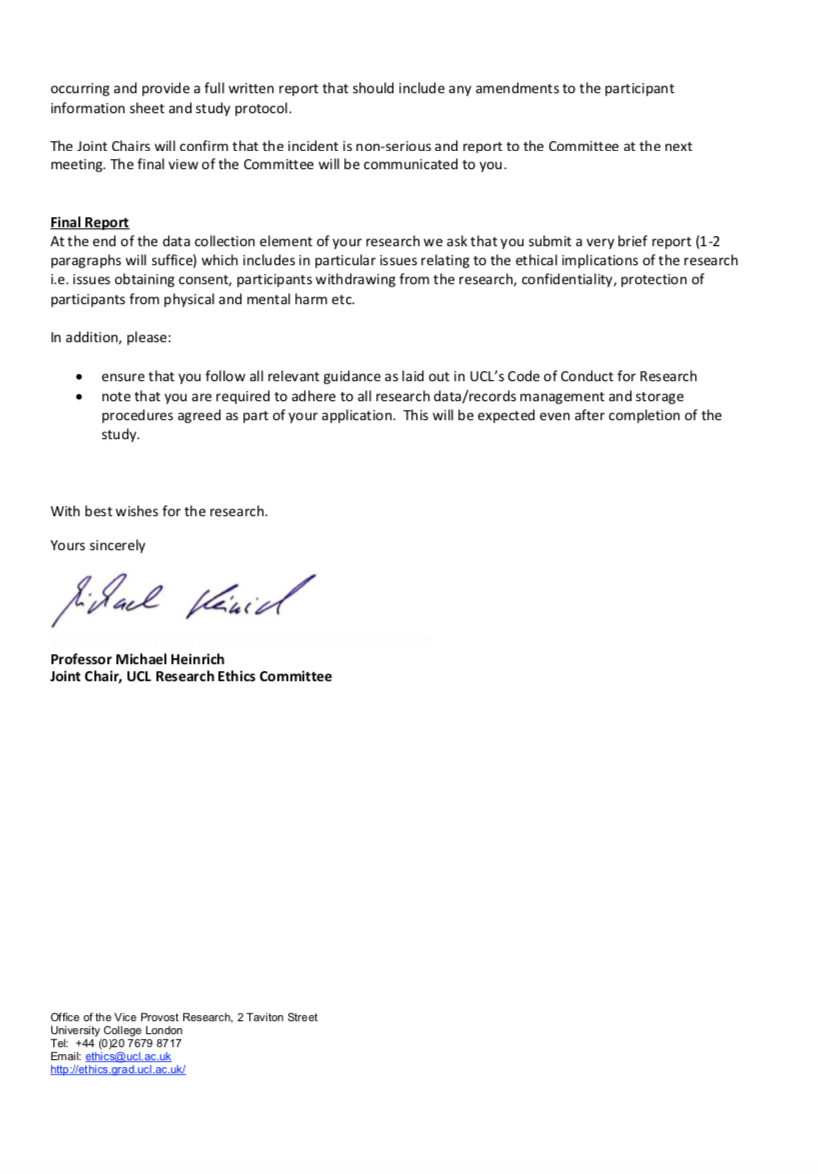


Supplementary material 2

Interview topic guide

**Research aims reminder (for interviewer only)**

To explore:

- Clinicians’ experience of contributing towards the treatment of young people with severe and complex eating disorder presentations in inpatient settings.
- Clinicians’ perspectives on how working in inpatient settings with severe and complex eating disorder presentations has interacted with their personal mental health and wellbeing.
- Clinicians’ perspectives on whether there are adequate resources in place to help them do their job well.

**Interview topic guide**

Hi, thank you for taking the time to talk with me today about your experiences of working in inpatient settings with severe and complex eating disorder presentations. During this interview I hope to discuss your experiences of working as a clinician who contributes to the treatment of severe and complex eating disorder presentations, and how this may have impacted on your mental health and wellbeing. I am also interested to find out about your thoughts about the struggles this group of service-users is facing and your views on what is needed in order to better support staff and service-users’ mental health and wellbeing. I have a standard list of questions I will be using to keep on track, but if at any time you do not feel comfortable talking about something that’s absolutely fine, just let me know – we can change the topic, take a break or stop the discussion at any point. **Do you have any questions before we get started?**

**Initial form(s) to complete:**

During the first stage of the interview, an initial icebreaker/warm-up conversation will be held. During this stage, the interviewer will complete a form with the participant which gathers data around their current general job title, role title, type of workplace and location. If participants have not had a chance to fill out the sociodemographic information form (which is attached to the consent form) prior to the interview, this will be completed with the participant at this point.

**Can you tell me about how you contribute towards the treatment of severe and complex eating disorder presentations in inpatient care?**

**Can you tell me about the presentations of the service-users you work with? Please maintain confidentiality.**

**Is there anything you find difficult in terms of treating this group of service-users?**

*Prompts:*

- *Is there anything else you find difficult?*

**Is there something you particularly enjoy about treating this group of service-users?**

*Prompts:*

- *Is there anything else you enjoy?*

**If you are comfortable doing so, can you tell me about some difficult experiences you have had in relation to treating this group of service-users?**

*Prompts:*

- *Upsetting events*
- *Challenging situations*
- *Any challenges related to the inpatient context*

**What helped you cope with these challenges?**

*Prompts:*

- *Emotionally*
- *Practically*
- *Interpersonally*

**How do you feel that treating this group of service-users has impacted you?**

*Prompts:*

- *In your personal life*
- *In your professional life*
- *Your wellbeing*

**What do you think can help clinicians maintain their well-being while working with this group of service users in an inpatient setting?**

*Prompts:*

- *In their personal life*
- *In their professional life*
- *Their wellbeing*

**Is there anything you would like to add before we finish?**
